# Quantitative Microvascular Change Analysis Using a Semi-Automated Software in Macula-off Rhegmatogenous Retinal Detachment Assessed by Swept-Source Optical Coherence Tomography Angiography

**DOI:** 10.3390/jcm13102835

**Published:** 2024-05-11

**Authors:** Pablo Díaz-Aljaro, Javier Zarranz-Ventura, Laura Broc-Iturralde, Nevena Romanic-Bubalo, Ignacio Díaz-Aljaro, Zhongdi Chu, Ruikang K. Wang, Xavier Valldeperas

**Affiliations:** 1 Department of Ophthalmology, Hospital Universitari Germans Trias i Pujol, 08916 Badalona, Spain; 2 Department of Surgery, Universitat Autònoma de Barcelona (UAB), 08193 Barcelona, Spain; 3 Department of Ophthalmology, Hospital Clínic de Barcelona, 08036 Barcelona, Spain; 4 Department of Ophthalmology, Hospital Clínico Universidad de Chile, Santiago 8380456, Chile; 5 Department of Bioengineering, University of Washington, Seattle, WA 98195, USA

**Keywords:** rhegmatogenous retinal detachment, microvascular changes, OCTA, macula off

## Abstract

**Objective**: To analyze the performance of custom semi-automated software for quantitative analysis of retinal capillaries in eyes with macula-off rhegmatogenous retinal detachment (RRD) and the role of these microvascular measures as potential biomarkers of postoperative visual outcomes. **Methods**: A prospective, observational, and single-center study was conducted on consecutive patients who underwent 25G pars-plana vitrectomy for primary uncomplicated macula-off RRD. Optical coherence tomography angiography (OCTA) was performed in the fellow and RRD eyes before surgery and in months 1, 3, and 6 after surgery. The preoperative values of the fellow eyes were used as surrogates of macula-off ones. The primary endpoints were the mean vessel diameter index (VDI); vessel area density (VAD); and vessel skeleton density (VSD) at month 6. **Results**: Forty-four eyes (44 patients) were included in the study. Considering the fellow eyes as a surrogate of preoperative values of macula-off eyes, VDI in superficial (SCP) and deep (DCP) capillary plexuses was significantly reduced at month 6 (*p* = 0.0087 and *p* = 0.0402, respectively); whereas VSD in SCP increased significantly from preoperative values (*p* = 0.0278). OCTA built-in software parameters were significantly reduced from month 1 to month 6 in both SCP and DCP (*p* values ranged between 0.0235 and <0.0001). At month 6, 25 (56.8%) eyes achieved a best-corrected visual acuity BCVA ≥ 0.3 (LogMAR). The greater the preoperative BCVA, the greater the probability of achieving good visual outcomes (Odds ratio: 11.06; *p* = 0.0037). However, none of the OCTA parameters were associated with the probability of achieving a BCVA improvement ≥ 0.3. **Conclusions**: Quantitative evaluation of capillary density and morphology through OCTA and semi-automated software represents a valuable tool for clinical assessment and managing the disease comprehensively.

## 1. Introduction

Rhegmatogenous retinal detachment (RRD) is the most common type of retinal detachment, with an incidence of 1 in 10,000 persons per year [1].

Despite the technical advances in retinal surgery, RRD is considered a sight-threatening condition and must be treated as quickly as possible [2,3,4]. Additionally, after having achieved a good anatomical retinal reattachment, the postoperative visual acuity (VA) gain is often incomplete in many subjects [1,2,3,4,5,6].

Optical coherence tomography (OCT) development has allowed the non-invasive assessment of the ultrastructural details of the retina. The preoperative status of the macula is one of the most important prognostic factors for visual recovery after surgical repair of RRD [7,8].

Optical coherence tomography angiography (OCTA) serves as a tool designed to provide both qualitative and quantitative analyzes of the retinal and choroidal microvasculature. Moreover, it enables examination of the macular microvasculature and its potential alterations due to RRD, which could impact visual outcomes following surgery [9,10,11]. OCTA technology is continuously evolving. Swept-source OCTA (SS-OCTA) technology uses longer wavelengths and higher scan speeds, which allow for increased penetration, larger scan areas, and improved image quality [11,12,13]. We present a custom semi-automated software designed to assess vessel morphology within SS-OCTA images in a layer-specific manner [14]. In a previous work, we evaluated these OCTA-derived microvascular changes in macula-on RRD [15] and observed that some preoperative values and their changes throughout time were predictors of visual outcomes.

The current study aimed to analyze this semi-automated software performance for quantitative analysis of retinal capillaries in eyes with macula off RRD. Additionally, we also assess the role of these microvascular measures as potential biomarkers of postoperative visual outcomes.

## 2. Methods

### 2.1. Study Design

A prospective, observational, single-center study was conducted on consecutive patients who underwent 25G pars-plana vitrectomy (PPV) for primary uncomplicated macula-off RRD between May 2020 and June 2021 at the Ophthalmology Department of Hospital Universitari Germans Trias i Pujol, Barcelona, Spain.

This study protocol was approved by the Ethics Committee of the Hospital Universitari Germans Trias i Pujol (OFT-AOCT-2018-01. Ref. CEI PI-18/159) and was conducted following the tenets of the Declaration of Helsinki and the Good Clinical Practice/International Council for Harmonization Guidelines. Written informed consent was obtained from all patients before participation in the study.

Any information that could lead to individual patient identification has been encrypted or removed, as appropriate, to guarantee their anonymity.

### 2.2. Study Participants

**Figure 1:**
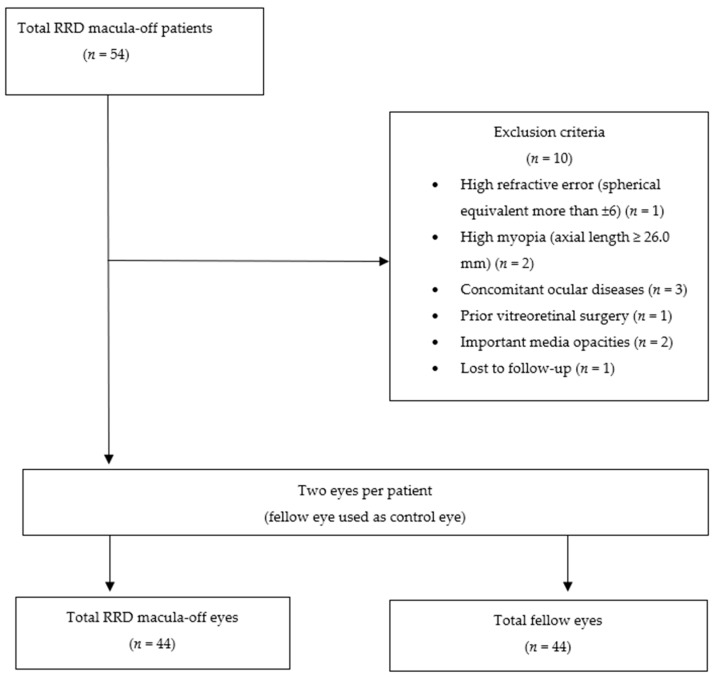
Flow chart showing the selection of patients in this study.

This study included consecutive patients, aged ≥18 years, with a clinical diagnosis of macula-off primary uncomplicated RRD (defined as a complete detachment of fovea centralis), who underwent a 25G PPV. The lens status of the patients (phakic and pseudophakic) did not interfere with the capture of good-quality images (Figure 1).

Patients were excluded if they had refractive errors (spherical equivalent) greater than ±6, high myopia (axial length ≥ 26.0 mm), concomitant ocular diseases (diabetic retinopathy, glaucoma, age-related macular degeneration, uveitis, retinal vascular disease, or epiretinal membrane in either eye), prior vitreoretinal surgery, prior retinal detachment in either eye, or important media opacities. Patients were also excluded if OCTA scans exhibited poor quality, indicated by an image quality score lower than 45 (Topcon Quality Index 0–100), and if incorrigible artifacts were present.

Patients underwent a complete ophthalmological examination of the affected eye (RRD eye) and the fellow eye, including best corrected visual acuity (LogMAR) and OCTA images (DRI OCT Triton; Topcon Corporation, Tokyo, Japan), before and at months 1, 3, and 6 after surgery. Axial length (AL) measurements were obtained using noncontact partial coherence laser interferometry (IOL Master 500, version 3.01; Carl Zeiss Meditec, Jena, Germany).

#### 2.2.1. Surgical Technique

All patients underwent pars plana vitrectomy (PPV), carried out with retrobulbar anesthesia by two different experienced high-volume surgeons (XV and LB). Standard three-port 25-gauge PPV without phacoemulsification was performed using the Alcon Constellation system (Alcon Laboratories, Inc., Fort Worth, TX, USA). no external limiting membrane peeling was performed, and SF6 and C3F8 served as tamponades. Retinal reattachment was confirmed by the complete disappearance of subretinal fluid and the flattening of retinal breaks post-gas reabsorption. Subsequent evaluations revealed no signs of macular edema, epiretinal membrane, or significant cataract development. 

#### 2.2.2. Optical Coherence Tomography Angiography

OCTA centered in the macula (DRI OCT Triton; Topcon Corporation, Tokyo, Japan) was performed in the fellow eyes before surgery and in both groups at months 1, 3, and 6 after surgery. An integrated eye-tracking system secured the positioning of follow-up scans. In cases of macula-off rhegmatogenous retinal detachment (RRD), conducting preoperative evaluations was not viable due to the compromised image quality caused by the detached retina. Consequently, we omitted instances where the distortion of the retinal structure impeded accurate segmentation.

For each eye, a 3 mm × 3 mm fovea-centered OCTA scan protocol was performed. The SS-OCT and OCTA images were interpreted using the web-based ophthalmic data management system (ImageNet 6 Version 1.20.11109; 2016, Topcon Corporation, Tokyo, Japan) and analysis was conducted on the two-dimensional superficial capillary plexus (SCP) and the deep capillary plexus (DCP) images. The images of the avascular plexus and choriocapillaris plexus were not considered for this study. Automatic segmentation of the superficial capillary plexus (SCP) and deep capillary plexus (DCP) was performed, and projection artifacts present in DCP were removed using the device’s built-in validated algorithm to improve the quality of the images [16]. Moreover, the foveal avascular zone (FAZ) area was also manually calculated. Central macular thickness (CMT) was assessed by the same OCT visualization platform using the retina map mode (in microns). The quantitative parameters analyzed using OCTA built-in software (OCTARA^TM^ (OCTA Ratio Analysis) Topcon Corporation, Tokyo, Japan) were vessel density (VD%) in the SCP and DCP, separated as foveal, parafoveal, and whole areas, and FAZ area (mm^2^).

**Figure 2:**
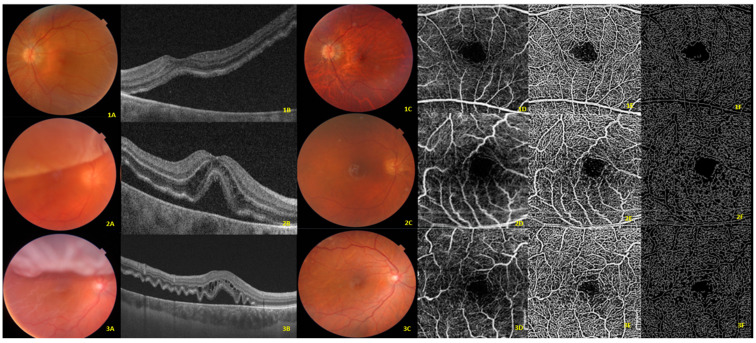
Visual illustrations that serve to depict the quantitative analysis algorithm in three cases at the superficial capillary plexus. (**1A**,**2A**,**3A**) Color fundus photography of macula-off rhegmatogenous retinal detachment at preoperative time. (**1B**,**2B**,**3B**) B-scan where a complete detachment of the fovea can be appreciated. (**1C**,**2C**,**3C**) Color fundus photography at postoperative month six with reattached retina. (**1D**,**2D**,**3D**) Displays the original optical coherence tomography angiography (OCTA) en-face image at postoperative month 6. (**1E**,**2E**,**3E**) Depicts the vessel area map at postoperative month 6, a binary vasculature image created through the Hessian filter and adaptive thresholding. This image is employed for the quantification of VAD and VDI. (**1F**,**2F**,**3F**) Represents the vessel skeleton map at postoperative month 6, generated by iteratively removing pixels from the outer perimeter of the vessel area map until only one pixel remains in the width direction of the vessels. This image is utilized in the quantification of VSD and VDI.

In addition, this study used custom semi-automated software [15,17] for evaluating vessel area density (VAD), vessel skeleton density (VSD), and vessel diameter index (VDI). To achieve this, the 2D grayscale En-Face SS-OCTA image was initially transformed into an 8-bit image (586 × 585 pixels) covering an area of 3 × 3 mm^2^ around the fovea (with 1 pixel equating to 5.13 × 5.13 µm^2^). Subsequently, a three-way approach comprising a global threshold, hessian filter, and adaptive threshold in MATLAB (R2013b; MathWorks, Inc., Natick, MA, USA) was employed to convert this image into a binary and skeletonized form. These parameters were initially intended to provide a quantitative assessment of capillary density (VAD and VSD) and morphology (VDI). VAD, which is calculated from the binary OCTA image, measures the proportion of the angiogram region that exhibits observable perfusion. By collapsing the cross-sectional width of each vessel segment into one pixel, the binarized image can be transformed into a skeletonized representation of the retinal vessels. VSD is then computed from this skeletonized image, and it denotes the absolute linear distance (length) of the blood vessels in the image. Unlike VAD, VSD represents the total length of the retinal vascular network, irrespective of vessel caliber [16,17,18,19]. In contrast, VDI is calculated from both binarized and skeletonized images, and it quantifies the mean vascular caliber (vessel diameter) (Figure 2). Previous studies have demonstrated the high reliability and repeatability of these parameters (VDI, VSD, and VAD) [18,19,20].

We have also assessed the correlation between the RRD eyes and the fellow eyes.

### 2.3. Main Outcomes

The primary endpoints were to establish significant variations in the mean VDI, VAD, and VSD from preoperatory to month 6 in the macula-off eyes.

Secondary endpoints included the differences in the foveal, parafoveal, whole area, FAZ, CMT, VDI, VAD, and VSD between the RRD eyes and the fellow eyes at the different time-point measures, and predictive factors associated with functional success (BCVA improvement ≥ 0.3 LogMAR at month 6).

For statistical purposes, the fellow eyes have been taken as a reference to establish the preoperative parameters of eyes with macula off RRD.

### 2.4. Statistical Analysis

The statistical analysis was performed with the MedCalc^®^ Statistical Software version 20.218 (MedCalc^®^ Software Ltd., Ostend, Belgium; https://www.medcalc.org; accessed on 13 May 2023).

Descriptive statistics, including mean ± standard deviation (SD), median (interquartile range, IqR), or number (percentage), were used as appropriate.

Data were tested for normal distribution using a Shapiro–Wilk test.

The Mann–Whitney U test was used to compare baseline and evolutive continuous variables, from preoperative to month 6, between study and fellow eyes.

A logistic regression model was used to estimate and test factors for their association with functional success. A backward strategy was adopted, with a statistically significant cut-off for variable screening of 0.05. Factors associated with BCVA gain ≥ 0.3 in the univariate analysis at *p* < 0.1 were included in the multivariate analysis.

Categorical variables were compared using a Chi-square test and a Fisher’s exact test, as appropriate. A *p* value of less than 0.05 was considered significant.

No formal sample size calculation was performed before initiating the study, as sufficient data were not available regarding the clinical performance of this software in this field. Consequently, the statistical significance observed regarding the outcome measures and their comparisons may be regarded as exploratory rather than confirmatory.

## 3. Results

### 3.1. Preoperative Demographic and Clinical Characteristics

Forty-four eyes from 44 patients were included in the study.

The mean age was 68.9 ± 11.8 years and 11 (25.0%) were women. A total of 25 (56.8%) eyes did not have preoperative PVR, 28 (63.7%) eyes had an RRD extension ≤ 2 quadrants, 23 (47.7%) were pseudophakic, and 21 were phakic (52.3%), which did not require subsequent cataract surgery during follow-up. The main demographics and clinical characteristics of the study population are presented in Table 1.

### 3.2. Semi-Automated Software Parameters

In the SCP of the fellow eyes, preoperative mean VDI, VAD, and VSD were 19.2 ± 0.1, 0.43 ± 0.00, and 0.16 ± 0.00, respectively. In the DCP, preoperative mean VDI, VAD, and VSD were 18.9 ± 0.1, 0.40 ± 0.00, and 0.15 ± 0.00 (Table 2).

Considering the fellow eyes as a surrogate of macula-off preoperative semi-automated software values, in RRD eyes the VDI in both SCP and DCP was significantly lower during the follow-up as compared to the preoperative values. Nevertheless, VSD in the SCP increased significantly from the preoperative values (Table 2).

On the contrary, if we do not consider the value of the fellow eye as a reference for preoperative values, there were no significant changes in any of the semi-automated software variables throughout the study (Table 2).

To assess the feasibility of using the measurements of the contralateral eye as a control, a study of the correlation of the semi-automated software metrics and the device built-in OCTA parameters at month 6 between the RRD eyes and their fellow eyes was performed (Table S1).

In the DCP, all the variables showed a significant correlation. However, in the SCP, only the VAD was significantly correlated.

**Figure 3:**
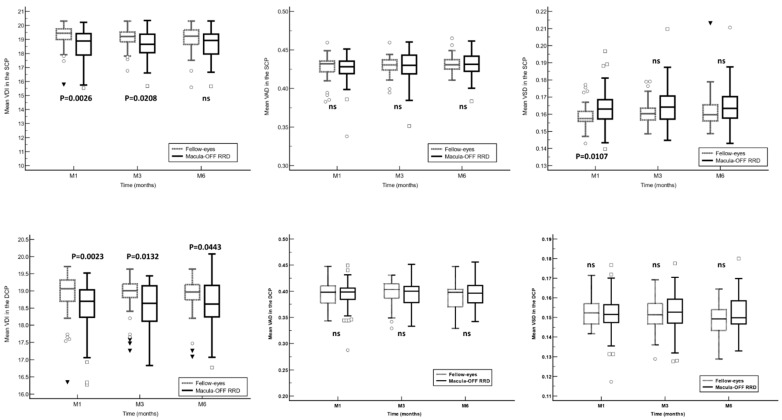
A comparison of the optical coherence tomography angiography semi-automated software variables in the superficial and deep capillary plexuses between the eyes with macula-off rhegmatogenous retinal detachment (RDD) and their fellow-eyes. Between-group comparisons were calculated with the Mann–Whitney U test. VDI: vessel density index; VAD: vessel area density; VSD: vessel skeleton density; SCP: superficial capillary plexus; DCP: deep capillary plexus; RDD: rhegmatogenous retinal detachment. Unfilled squares: Measurements that exceed the value of the 95% percentile. Unfilled circles: Measurements that exceed the value of the 97.5% percentile. Black filled triangles: Measurements that exceed the value of the 99% percentile.

In the SCP, VDI was significantly lower in RRD eyes at months 1 and 3, although there were no significant differences at month 6. Similarly, in the DCP, VDI was significantly lower in RRD eyes at all follow-up time points measured. The VSD in the SCP was significantly lower in the eyes with macula off RRD at month 1 (Figure 3).

### 3.3. OCTA Built-In Software Parameters

An overview of the preoperative and follow-up OCTA built-in software parameters, in both SCP and DCP, is shown in Table 3. Compared with the values obtained one month after surgery, the OCTA parameters in the SCP, DCP, and CMT showed a significant reduction at month 6, whereas the FAZ area showed an increase (Table 3).

Different parameters throughout the follow-up of the study, both in the SCP and in the DCP, have shown significant differences between eyes with macula off RRD and fellow eyes (Figure S1). To further investigate the impact of preoperative vascular status on the visual outcomes, we performed a comparison between eyes that achieved a BCVA gain ≥ 0.3 (LogMAR) and those that did not. Although a certain tendency has been observed to find better preoperative retinal hemodynamic parameters in eyes that achieved an improvement in BCVA ≥ 0.3 (LogMAR), this difference has not been statistically significant (Table S2).

### 3.4. Best Corrected Visual Acuity

In the eyes with macula-off RRD, there was a statistically linear progression from preoperative to month 6 values (mean difference: −0.32 ± 0.36; 95% CI: −0.43 to −0.21; *p* < 0.0001).

In the RRD eye group at month 6, 6 (13.6%) eyes had BCVA loss ≥ 0.1 (LogMAR) as compared to preoperative values; while 28 (63.6%) eyes achieved a BCVA improvement ≥ 0.1 (LogMAR) as compared to preoperative values (Table S3).

### 3.5. Predictive Factors for Functional Outcome

At month 6, 25 (56.8%) eyes had achieved a BCVA gain ≥ 0.3 (LogMAR).

Preoperative BCVA was significantly associated with the probability of achieving a BCVA improvement ≥ 0.3 (LogMAR). The greater the preoperative BCVA, the greater the probability of achieving good visual outcomes (Odds ratio: 11.06; 95% confidence interval: 7.45 to 19.3; *p* = 0.0037).

However, none of the other variables analyzed showed any relationship with the probability of achieving a BCVA improvement ≥ 0.3 (LogMAR) (Table 4).

## 4. Discussion

The current study evaluated the superficial and deep capillary plexuses using two different OCTA measurement systems, as well as their association with the visual acuity outcomes in patients with RRD with macular involvement after surgery. There is a well-established understanding that foveal microvascular parameters like FAZ and VD exhibit significant variability among subjects. To counteract this variability, we chose to analyze the fellow eye as the control [21]. In studies where the contralateral eye is not utilized as the control, various authors have observed differences that could be solely attributed to interindividual variations.

This study did not observe significant changes in any of the semi-automated software parameters analyzed, either SCP or DCP, throughout the follow-up. However, as compared to the fellow-eyes, the VDI in the SCP and DCP showed significantly lower values in the RRD eyes throughout the study follow-up. Similarly, the OCTA built-in commercial software parameters tend to show significantly lower values in the RRD eyes than in the fellow eyes.

In this study, preoperative BCVA was significantly associated with the probability of achieving good visual outcomes. However, none of the OCTA-derived retinal hemodynamic parameters was significantly associated with the visual outcomes.

Making an accurate prediction of the postoperative best-corrected visual acuity (BCVA) in eyes with macula-off retinal detachment (RRD) is a challenging task. While various factors have been linked to the likelihood of achieving improved postoperative visual outcomes [22,23,24], the preoperative BCVA has been identified as a significant predictor of postoperative visual recovery following macula-off RRD surgery [24,25,26]. We obtained similar results from our study. Preoperative BCVA may be affected by several factors, including the retinal hemodynamic status or the integrity of the retinal microstructures [24,27]. A previous study by Chatziralli et al. [28] demonstrated that in macula-off detachments, a duration longer than one week was found to be a detrimental prognostic factor for visual outcome. Our results, conducted through both univariate and multivariate methods, did not find evidence to support this correlation. Moreover, retinal, and choroidal vascular impairment secondary to RRD leads to reduced retinal perfusion, causing macular ischemia, which eventually hampers visual acuity improvement [27].

Recent studies using OCTA revealed that macular capillary perfusion was damaged even after macular reattachment [29,30,31]. McKay et al. [31] reported lower vessel density in the DCP of macula off RRD eyes as compared to the fellow healthy eyes, which was correlated with worse visual acuity.

Woo et al. [32] reported that FAZ was negatively correlated with BCVA after RRD repair in both macula-on and macula-off eyes. Machairoudia et al. [33] found significant changes in the foveal avascular zone in the superficial and deep capillary plexus at 6 months between operated eyes compared to fellow eyes with moderate correlation with BCVA, and no significant differences in vessel density were observed.

However, our study did not confirm these findings. In fact, we did not find any relationship between any of the OCTA parameters (either SCP or DCP) and the visual outcomes at month 6. These results are in line with those published by Sato et al. [34], who reported that the FAZ area had no correlation with postoperative BCVA after vitrectomy in macula off RRD.

Our study found that the VDI in the DCP was significantly lower in RRD eyes compared with the fellow eyes throughout the study follow-up at all time points, but in the SCP only at months 1 and 3. VDI stands for the mean vessel caliber, which acts as a morphological indicator enabling us to infer the dynamic alterations occurring in the macular microvasculature during detachment. An increase in vascular congestion induced by photoreceptor ischemia associated with detachment, followed by capillary contraction, lends support to this hypothesis. Piccolino et al. [35] observed capillary dilation and hyperpermeability, a response to tissue hypoxia, in 50 eyes studied using fluorescein angiography, further validating this concept. Agarwal et al. [36] also reported a significant enlargement in the FAZ area alongside a decrease in VD and fractal dimension in the SCP and DCP. The latter measures the intricacy of the vascular microarchitecture, providing additional evidence of these evolving changes using another novel analysis technique. Regarding in-built software parameters, we found a decrease in the mean VD (foveal, parafoveal, and whole) and CMT, and an increase in mean FAZ area in both the SCP and DCP compared to fellow eyes at postoperative month 6. Our results are in line with other researchers who have noted a reduction in VD, mainly in the DCP, and an expansion of the FAZ area [31,34,36]. Çetinkaya-Yaprak et al. [37] also demonstrated an increase in the FAZ area and a decrease in retinochoroidal flow within the initial three months post-surgery.

Located in the watershed zone, the DCP may be more susceptible to ischemia after detachment due to its lower oxygen concentration and lower perfusion pressure. This could partially explain why eyes with macula-off RRD obtain significantly lower postoperative visual acuities than macula-on eyes [38].

Conversely, Wang et al. [39] investigated retinal microcirculation during the first three months after surgery, reporting a gradual increase in VD and recovery of macular perfusion, with no difference noted in the contralateral healthy eye at the end of follow-up. They suggested a rehabilitation curve for different retinal plexuses, indicating a gradual recovery of macular vascularization with the attached and flattened retina. We did find that mean VSD (the absolute vessel length in the entire scan) at the SCP increased significantly from the preoperative values.

In eyes with RRD, different vascular and inflammatory coexisting events can lead to the activation of Müller cells, causing alterations in retinal blood flow. These events induce structural changes that may alter retinal ganglion cells and photoreceptors functionality, with the subsequent lack of visual improvement [40,41]. It might be hypothesized that hypoxia and secondary release of inflammatory biomarkers (vasoconstrictor endothelin 1) after neurosensory detachment of the retinal pigment epithelium would lead to retinal blood flow impairment. However, the confirmation of this hypothesis falls beyond the scope of the current study.

Our study, despite some limitations, yielded valuable insights. While it was conducted at a single center with a relatively modest sample size and a brief follow-up period, mirroring the scope of many surgical studies in this domain, it is important to highlight that we examined 44 eyes affected by macula off RRD. This sample size surpasses that of comparable studies [31,32,33,34,35,36,37,38,39]. Inherent to the studied pathology, the inability to determine the preoperative OCTA parameters in eyes with macula off RRD limited the metrics comparison after surgery. Although measurements of fellow eyes could have served as controls for the RRD eyes, as suggested by Hong et al. [38], the lack of correlation in the VDI, VSD, foveal, and whole in the SCP made us exercise caution when interpreting the results related to the predictive value of changes in the described parameters. We also acknowledge the presence of inter-eye differences, particularly in systemic diseases. The considerable variation among patients in terms of factors like proliferative vitreoretinopathy (PVR) and retinal detachment extent highlights the heterogeneity of the study group. This diversity poses a potential challenge, as it could introduce bias into the data.

In conclusion, changes in macular microvascularization after successful RRD repair remain ambiguous. Despite noticeable visual changes in eyes following RRD repair, we were unable to identify specific OCTA biomarkers, and the small patient sample size prevented us from forming distinct subgroups based on visual acuity. The challenge of detecting changes that could predict functional recovery remains a significant concern for retinal surgeons. The affected eyes exhibited a lower vascular density index (VDI) in both superficial and deep capillary plexuses compared to the fellow eyes over the course of the study. The most significant factor associated with the likelihood of achieving favorable visual outcomes was the preoperative BCVA. The use of OCTA and this semi-automated software not only facilitated the characterization of retinal microvascular circulation and its changes during the follow-up period in eyes with macula off RRD, but also contributed to reinforcing the observable changes with the classic parameters already included in the in-built software. Together, these new markers and established parameters have the potential to enhance overall performance and sensitivity in assessing vascular alterations and may serve as a valuable measure for clinical evaluation, treatment response, and overall disease management.

## Figures and Tables

**Table 1:** Overview of the main demographic and clinical characteristics of the study population.

	Study Sample (*n* = 44)
Age, years	
Mean ± SD	68.9 ± 11.8
Median (IqR)	68.0 (62.3 to 77.1)
Sex, *n* (%)	
Women	11 (25.0)
Men	33 (75.0)
Systemic diseases, *n* (%)	
DM	13 (29.5)
HBP	22 (50.0)
Dyslipidemia	22 (50.0)
Smoker	5 (12.2)
Other	13 (29.5)
Eye, *n* (%)	
Right	23 (52.3)
Left	21 (47.7)
PVR, *n* (%)	
No	25 (56.8)
Degree A	7 (15.9)
Degree B	9 (20.5)
Degree C	3 (6.8)
Time, *n* (%)	
<7 days	28 (63.6)
≥7 days	16 (36.4)
Extension, *n* (%)	
1 quadrant	1 (2.3)
2 quadrants	27 (61.4)
3 quadrants	7 (15.9)
4 quadrants	9 (20.5)
Lens status, *n* (%)	
Phakia	21 (47.7)
Pseudophakia	23 (52.3)
IOP, mmHg	
Mean ± SD	16.4 ± 3.3
Median (IqR)	16.0 (16.0 to 18.0)
Axial length, mm	
Mean ± SD	24.9 ± 1.6
Median (IqR)	24.9 (23.8 to 26.0)
BCVA, logMAR	
Mean ± SD	0.83 ± 0.29
Median (IqR)	1.00 (0.70 to 1.00)
CMT ^⁂^, µm	
Mean ± SD	288.0 ± 25.5
Median (IqR)	283.0 (275.5 to 304.0)

^⁂^ Information of the fellow eyes. SD: standard deviation; IqR: interquartile range; DM: diabetes Mellitus; HBP: systemic high blood pressure; PVR: proliferative vitreoretinopathy; IOP: intraocular pressure; BCVA: best corrected visual acuity; CMT: central macular thickness.

**Table 2:** Overview of the preoperative and follow-up semi-automated software parameters in the superficial capillary and deep capillary plexuses in the eyes with macula-off rhegmatogenous retinal detachment (RRD). Statistical significance was calculated with the repeated analysis of variance (ANOVA) test.

	Superficial Capillary Plexus (*n* = 44)				
	Preoperative *	Month 1	Month 3	Month 6	*p*
VDI					
Mean ± SE	19.2 ± 0.1	18.6 ± 0.2	18.6 ± 0.2	18.7 ± 0.2	0.0087 ^a^
95% CI	19.0–19.4	18.3–18.9	18.3–18.9	18.3–19.0	0.9649 ^b^
VAD					
Mean ± SE	0.43 ± 0.00	0.43 ± 0.00	0.43 ± 0.00	0.43 ± 0.00	0.2729 ^a^
95% CI	0.42–0.43	0.43–0.44	0.43–0.44	0.42–0.43	0.1854 ^b^
VSD					
Mean ± SE	0.16 ± 0.00	0.17 ± 0.00	0.17 ± 0.00	0.16 ± 0.00	0.0278 ^a^
95% CI	0.16–0.16	0.16–0.17	0.16–0.17	0.16–0.17	0.5748 ^b^
	Deep capillary plexus (*n* = 44)				
	Preoperative *	Month 1	Month 3	Month 6	*p*
VDI					
Mean ± SE	18.9 ± 0.1	18.5 ± 0.2	18.7 ± 0.1	18.6 ± 0.1	0.0402 ^a^
95% CI	18.7–19.1	18.2–18.8	18.5–19.0	18.4.18.9	0.2926 ^b^
VAD					
Mean ± SE	0.40 ± 0.00	0.40 ± 0.01	0.40 ± 0.01	0.39 ± 0.01	0.5564 ^a^
95% CI	0.39–0.40	0.39–0.41	0.38–0.41	0.38–0.40	0.6508 ^b^
VSD					
Mean ± SE	0.15 ± 0.00	0.15 ± 0.00	0.15 ± 0.00	0.15 ± 0.00	0.5210 ^a^
95% CI	0.15–0.15	0.15–0.16	0.15–0.16	0.15–0.15	0.9003 ^b^

* Fellow eyes have been taken as reference to establish the preoperative parameters. ^a^ Considering fellow eye parameters as reference to establish the preoperative values. ^b^ Without considering fellow eye parameters as reference to establish the preoperative values. SE: standard error; 95% CI: 95% confidence interval; VDI: vessel diameter index; VAD: vessel area density; VSD: vessel skeleton density.

**Table 3:** Overview of the preoperative and follow-up optical coherence tomography angiography (OCTA) built-in software parameters in the superficial capillary and deep capillary plexuses in the eyes with macula-off rhegmatogenous retinal detachment (RRD). Statistical significance was calculated with the repeated analysis of variance (ANOVA) test.

	Fellow Eyes (*n* = 44 Eyes)							
	FSCP	PFSCP	WSCP	FDCP	PFDCP	WDCP	FAZ	CMT
Preoperative								
Mean ± SE	20.9 ± 0.7	48.3 ± 0.3	42.8 ± 0.3	20.8 ± 1.1	56.7 ± 0.4	49.5 ± 0.4	0.24 ± 0.02	288.0 ± 3.8
95% CI	19.6 to 22.3	47.7 to 48.9	42.3 to 43.4	18.5 to 23.0	55.8 to 57.6	48.8 to 50.3	0.21 to 0.27	280.3 to 295.8
Month 1								
Mean ± SE	20.8 ± 0.7	48.4 ± 0.3	42.9 ± 0.3	20.9 ± 1.1	56.7 ± 0.5	49.5 ± 0.4	0.24 ± 0.02	287.5 ± 3.9
95% CI	19.4 to 22.2	47.7 to 49.1	42.3 to 43.5	18.8 to 23.1	55.7 to 57.6	48.8 to 50.3	0.22 to 0.27	279.7 to 295.3
Month 3								
Mean ± SE	21.0 ± 0.7	48.4 ± 0.3	42.9 ± 0.3	20.9 ± 1.1	56.7 ± 0.5	49.5 ± 0.4	0.25 ± 0.01	287.6 ± 4.0
95% CI	19.6 to 22.3	47.8 to 49.1	42.4 to 43.5	18.7 to 23.1	55.7 to 57.6	48.8 to 50.3	0.22 to 0.27	279.5 to 295.7
Month 6								
Mean ± SE	20.9 ± 0.7	48.4 ± 0.3	42.9 ± 0.3	21.0 ± 1.1	56.7 ± 0.5	49.5 ± 0.4	0.24 ± 0.01	285.2 ± 4.4
95% CI	19.6 to 22.3	47.8 to 49.1	42.3 to 43.5	18.8 to 23.2	55.8 to 57.6	48.7 to 50.4	0.22 to 0.27	276.3 to 294.1
Intragroup Significance	0.6932	0.3363	0.3367	0.1053	0.8919	0.7830	0.6644	0.2845
	RRD eyes (*n* = 44 eyes)							
	FSCP	PFSCP	WSCP	FDCP	PFDCP	WDCP	FAZ	CMT
Preoperative *								
Mean ± SE	20.9 ± 0.7	48.3 ± 0.3	42.8 ± 0.3	20.8 ± 1.1	56.7 ± 0.4	49.5 ± 0.4	0.24 ± 0.02	288.0 ± 3.8
95% CI	19.6 to 22.3	47.7 to 48.9	42.3 to 43.4	18.5 to 23.0	55.8 to 57.6	48.8 to 50.3	0.21 to 0.27	280.3 to 295.8
Month 1								
Mean ± SE	22.9 ± 0.9	47.4 ± 0.3	42.5 ± 0.3	21.3 ± 0.7	55.4 ± 0.6	48.6 ± 0.4	0.25 ± 0.01	292.3 ± 6.6
95% CI	21.0 to 24.8	46.8 to 48.1	41.9 to 43.1	19.9 to 22.6	54.3 to 56.5	47.7 to 49.5	0.23 to 0.28	278.9 to 305.7
Month 3								
Mean ± SE	21.8 ± 0.7	46.5 ± 0.3	41.6 ± 0.3	20.5 ± 0.7	54.1 ± 0.6	47.4 ± 0.5	0.26 ± 0.01	269.3 ± 6.4
95% CI	20.4 to 23.3	45.9 to 47.2	41.1 to 42.1	19.2 to 21.8	52.8 to 55.4	46.4 to 48.4	0.23 to 0.28	256.3 to 282.3
Month 6								
Mean ± SE	20.0 ± 0.7	47.8 ± 0.4	40.8 ± 0.3	20.0 ± 0.7	52.9 ± 0.7	46.3 ± 0.6	0.26 ± 0.01	258.3 ± 6.4
95% CI	18.6 to 21.3	45.0 to 46.6	40.2 to 41.4	18.6 to 21.3	51.4 to 54.3	45.1 to 47.4	0.24 to 0.28	245.5 to 271.1
Intragroup Significance ^a^	0.0459	<0.0001	<0.0001	0.3006	<0.0001	<0.0001	0.0194	<0.0001
Intragroup Significance ^b^	0.0052	<0.0001	<0.0001	<0.0001	<0.0001	<0.0001	0.0235	<0.0001

* Fellow eyes have been taken as reference to establish the preoperative parameters of the OCTA. ^a^ Considering the fellow eyes OCTA built-in software parameters as reference to establish the preoperative values. ^b^ Without considering fellow eyes OCTA built-in software parameters as reference to establish the preoperative values. Statistical significance was calculated with the values of months 1, 3, and 6. SE: standard error; 95% CI: 95% confidence interval; FSCP: foveal superficial capillary plexus; PFSCP: parafoveal superficial capillary plexus; WSCP: whole superficial capillary plexus; FDCP: foveal deep capillary plexus; PFDCP: parafoveal deep capillary plexus; WDCP: whole deep capillary plexus; FAZ: foveal avascular zone; CMT: central macular thickness.

**Table 4:** Univariate and multivariate analysis to evaluate the potential factors for achieving a best corrected visual acuity improvement ≥ 0.3 in RRD eyes at month 6. Variables with a *p* < 0.1 in the univariate analysis were included in the multivariate analysis.

	Study Sample (*n* = 44)	
Variable	OR (95% CI)	*p*
Age ^a^	1.02 (0.97 to 1.07)	0.5104
Sex		
Ref Men		
Women	1.29 (0.32 to 5.28)	0.7236
DM		
Ref No		
Yes	1.85 (0.47 to 7.32)	0.3789
HBP		
Ref No		
Yes	1.46 (0.44 to 4.88)	0.5404
Dyslipidemia		
Ref No		
Yes	1.46 (0.44 to 4.88)	0.5404
RD extension		
Ref 1 quadrant		
≥2 quadrants	1.86 (0.54 to 6.41)	0.3279
Time		
Ref < 7 days		
≥7 days	0.83 (0.24 to 2.89)	0.7721
PVR		
Ref No		
Yes	1.35 (0.40 to 4.57)	0.6328
Axial length ^b^	1.01 (0.53 to 1.91)	0.9853
Preop BCVA	11.6 (7.45 to 19.3)	0.0037
Preop VDI SCP ^c,1^	0.56 (0.21 to 1.49)	0.2451
Preop VAD SCP ^d,1^	0.48 (0.23 to 3.94)	0.5820
Preop VSD SCP ^d,1^	1.83 (0.69 to 4.81)	0.6182
Preop VDI DCP ^c,1^	1.20 (0.40 to 3.59)	0.7470
Preop VAD DCP ^d,1^	0.43 (0.09 to 2.78)	0.3058
Preop VSD DCP ^d,1^	0.50 (0.21 to 1.23)	0.2428
Mean change ^2^ VDI SCP ^e^	1.10 (0.61 to 1.99)	0.7480
Mean change ^2^ VAD SCP ^d^	1.55 (0.09 to 2.62)	0.4255
Mean change ^2^ VSD SCP ^d^	1.63 (0.94 to 2.71)	0.7669
Mean change ^2^ VDI DCP ^e^	0.96 (0.41 to 2.25)	0.9326
Mean change ^2^ VAD DCP ^d^	1.21 (0.24 to 1.68)	0.3263
Mean change ^2^ VSD DCP ^d^	1.28 (0.60 to 2.72)	0.3507
Preop FSCP ^c,1^	1.05 (0.92 to 1.20)	0.4818
Preop PFSCP ^c,1^	1.21 (0.83 to 1.76)	0.3230
Preop WSCP ^c,1^	1.32 (0.85 to 2.05)	0.2168
Preop FDCP ^c,1^	1.04 (0.94 to 1.14)	0.4803
Preop PFDCP ^c,1^	1.16 (0.93 to 1.46)	0.1885
Preop WDCP ^c,1^	1.25 (0.95 to 1.66)	0.1099
FAZ	0.06 (0.00 to 32.67)	0.3851
CMT	1.02 (0.99 to 1.04)	0.2624
Mean change FSCP ^3^	2.48 (0.68 to 9.04)	0.1685
Mean change PFSCP ^3^	0.78 (0.38 to 1.58)	0.4833
Mean change WSCP ^3^	0.84 (0.37 to 1.88)	0.6629
Mean change FDCP ^3^	0.69 (0.31 to 1.54)	0.3628
Mean change PFDCP ^3^	1.06 (0.44 to 2.53)	0.8961
Mean change WDCP ^3^	0.93 (0.35 to 2.48)	0.8859
Mean change FAZ ^3^	0.94 (0.42 to 3.07)	0.6551
Mean change CMT ^3,f^	1.03 (0.99 to 1.07)	0.1617

^a^ Per year older. ^b^ Per mm longer. ^c^ Per unit greater. ^d^ Per 0.01 units greater. ^e^ Per 0.1 unit greater. ^f^ Per µm greater. ^1^ Considering the fellow eye parameters as reference to establish the preoperative values. ^2^ Mean change from month 1 to month 6 values. ^3^ Mean change from preoperative to month 6 values. DM: diabetes mellitus; HBP: high blood pressure; RD: retinal detachment; PVR: proliferative vitreoretinopathy; Preop: preoperative; BCVA: best corrected visual acuity; SCP: superficial capillary plexus; DCP: deep capillary plexus; VDI: vessel diameter index; VAD: vessel area density; VSD: vessel skeletal density; FSCP: foveal superficial capillary plexus; PFSCP: parafoveal superficial capillary plexus; FDCP: foveal deep capillary plexus; PFDCP: parafoveal deep capillary plexus; WDCP: whole deep capillary plexus; FAZ: foveal avascular zone; CMT: central macular thickness.

## Data Availability

Data available upon request from the corresponding author.

## Supplementary Materials

The following supporting information can be downloaded at: https://www.mdpi.com/article/10.3390/jcm13102835/s1, Table S1. Spearman rank correlation coefficient (rho) of the optical coherence tomography angiography (semi-automated and OCTA software) parameters of RRD eyes and their fellow eyes at month 6. Table S2. A comparison of the preoperative * optical coherence tomography angiography (OCTA) built-in software parameters between the eyes that achieved a best corrected visual acuity (BCVA) gain ≥ 0.3 and those that did not. *p* values were calculated with the Mann–Whitney U test. Table S3. Percentage of eyes that experienced different changes in best corrected visual acuity (BCVA) regardless of the preoperative BCVA. Figure S1. A comparison of the optical coherence tomography angiography (OCTA) built-in software parameters variables, central macular thickness (CMT), and foveal avascular zone (FAZ) in the superficial and deep capillary plexuses between the eyes with macula-off rhegmatogenous retinal detachment (RDD) and their fellow eyes. Between-group comparisons were calculated with the Mann–Whitney U test. FSCP: foveal superficial capillary plexus; PFSCP: parafoveal superficial capillary plexus; WSCP: whole superficial capillary plexus; FDCP: foveal deep capillary plexus; PFDCP: parafoveal deep capillary plexus; WDCP: whole deep capillary plexus; FAZ: foveal avascular zone; CMT: central macular thickness.
