# Supplementary material for: Quantitative Microvascular Change Analysis Using a Semi-Automated Software in Macula-off Rhegmatogenous Retinal Detachment Assessed by Swept-Source Optical Coherence Tomography Angiography
Source: J Clin Med. 2024 May 11;13(10):2835. doi: 10.3390/jcm13102835 (PMC11122367; doi:10.3390/jcm13102835)
Supplement: Supplementary file 1 [file jcm-13-02835-s001.zip › jcm-2981548-supplementary.pdf]

Table S1. Spearman rank correlation coefficient (rho) of the optical coherence tomography angiography (semi-automated and OCTA software) parameters of RRD eyes and their fellow eyes at month 6.

	Semi-automated software	
	SCP	DCP
VDI<br>Rho (95%CI)<br>p	 0.185 (-0.118 to 0.457)<br>0.2284	 0.337 (0.045 to 0.576)<br>0.0253
VAD<br>Rho (95%CI)<br>p	 0.463 (0.192 to 0.668)<br>0.0016	 0.410 (0.129 to 0.630)<br>0.0057
VSD<br>Rho (95%CI)<br>p	 0.202 (-0.099 to 0.472)<br>0.1848	 0.404 (0.122 to 0.626)<br>0.0066
	OCTA built-in software	
	SCP	DCP
Foveal<br>Rho (95%CI)<br>p	 0.457 (0.186 to 0.664)<br>0.0018	 0.597 (0.365 to 0.759)<br><0.0001
Parafoveal<br>Rho (95%CI)<br>p	 0.161 (-0.143 to 0.437)<br>0.2970	 0.430 (0.153 to 0.645)<br>0.0036
Whole<br>Rho (95%CI)<br>p	 0.024 (-0.275 to 0.319)<br>0.8773	 0.412 (0.131 to 0.632)<br>0.0054
FAZ<br>Rho (95%CI)<br>p	 0.917 (0.852 to 0.954)<br><0.0001	
CMT<br>Rho (95%CI)<br>p	 0.520 (0.264 to 0.708)<br>0.0003	

VDI: Vessel diameter index; VAD: Vessel area density; VSD: Vessel skeleton density; SCP: Superficial capillary plexus; DCP: Deep capillary plexus; FAZ: Foveal avascular zone; CMT: Central macular thickness.

Table S2. A comparison of the preoperative* optical coherence tomography angiography (OCTA) built-in software parameters between the eyes that achieved a best corrected visual acuity (BCVA) gain ≥0.3 and those that did not. P values were calculated with the Mann-Whitney U test.

	BCVA gain <0.3	BCVA gain ≥0.3	Difference (95% CI)**	p
FSCP, mean±SD	20.4±3.6	21.3±5.1	-1.1 (-4.0 to 1.8)	0.4102
PFSCP, mean±SD	47.9±1.3	48.5±2.3	-0.3 (-1.4 to 0.7)	0.4888

WSCP, mean±SD	42.4±1.1	43.1±2.1	-0.5 (-1.4 to 0.5)	0.3397
FDCP, mean±SD	19.8±3.3	21.4±9.1	-0.6 (-4.0 to 3.0)	0.6078
PFDCP, mean±SD	56.0±3.0	57.2±2.8	-1.2 (-3.2 to 0.6)	0.2827
WDCP, mean±SD	48.8±2.2	50.1±2.6	-1.0 (-2.6 to 0.4)	0.1736
FAZ, mean±SD	0.26±0.10	0.23±0.10	0.04 (-0.04 to 0.10)	0.2935
CMT, mean±SD	282.8±28.7	291.7±22.9	-6.0 (-20.0 to -6.0)	0.3579

* Preoperative parameters of fellow eyes have been considered. **Hodges-Lehmann median difference. BCVA: Best corrected visual acuity; SD: Standard deviation; 95%CI: 95% confidence interval; FSCP: Foveal superficial capillary plexus; PFSCP: Parafoveal superficial capillary plexus; WSCP: whole superficial capillary plexus; FDCP: Foveal deep capillary plexus; PFDCP: Parafoveal deep capillary plexus; WDCP: whole deep capillary plexus; FAZ: Foveal avascular zone; CMT: Central macular thickness.

Table S3. Percentage of eyes that experienced different changes in best corrected visual acuity (BCVA) regardless of the preoperative BCVA.

	RRD eyes
BCVA loss ≥ 0.3, n (%)	4 (9.1)
BCVA loss ≥ 0.2, n (%)	4 (9.1)
BCVA loss ≥ 0.1, n (%)	6 (13.6)
BCVA no changed, n (%)	10 (22.7)
BCVA gain ≥ 0.1, n (%)	28 (63.6)
BCVA gain ≥ 0.2, n (%)	27 (61.4)
BCVA gain ≥ 0.3, n (%)	25 (56.8)

BCVA: Best corrected visual acuity.

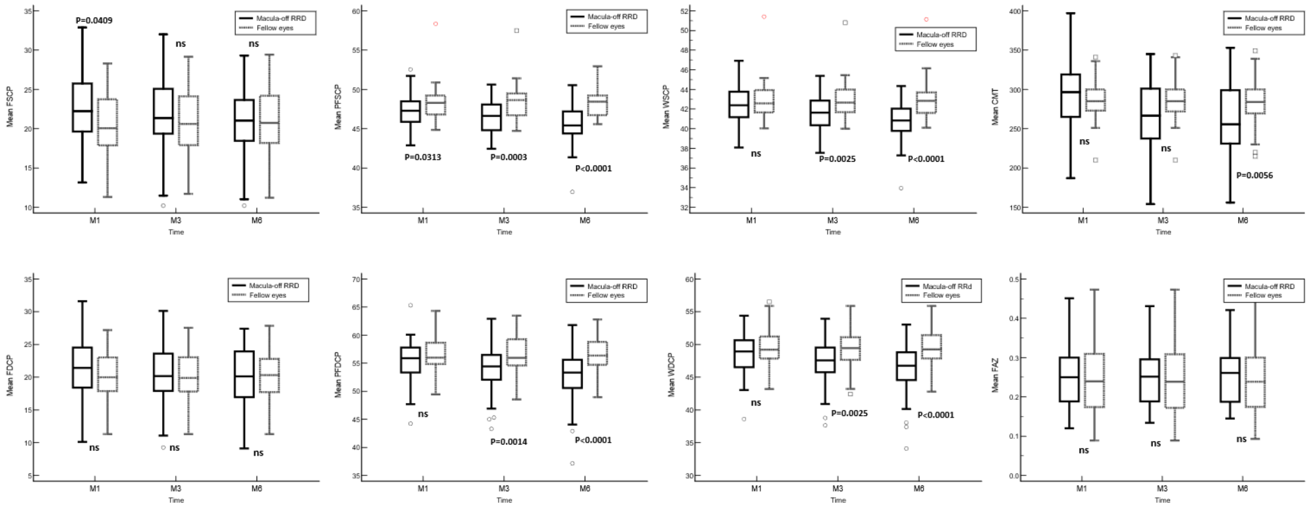

Figure S1. A comparison of the optical coherence tomography angiography (OCTA) built-in software parameters variables, central macular thickness (CMT) and foveal avascular zone (FAZ) in the superficial and deep capillary plexuses between the eyes with macula off rhegmatogenous retinal detachment (RDD) and their fellow eyes. Between group comparisons were calculated with the Mann-Whitney U test. FSCP: Foveal superficial capillary plexus; PFSCP: Parafoveal superficial capillary plexus; WSCP: whole superficial capillary plexus; FDCP: Foveal deep capillary plexus; PFDCP: Parafoveal deep capillary plexus; WDCP: whole deep capillary plexus; FAZ: Foveal avascular zone; CMT: Central macular thickness.
